# A Case of Primary Central Nervous System Lymphoma Mimicking a Demyelinating Disorder: From Steroids to Stem Cell Transplant

**DOI:** 10.7759/cureus.95037

**Published:** 2025-10-21

**Authors:** Sumana Reddy Tanigundala, Vishesh Anil Agrawal, Ravikrishna Madala

**Affiliations:** 1 Medical Oncology, KIMS-Sunshine Hospital, Hyderabad, IND; 2 Radiology, KIMS-Sunshine Hospital, Hyderabad, IND

**Keywords:** autologous hematopoietic stem cell transplantation, cns lymphoma, demyelinating neurological disorder, mimick, steroid

## Abstract

Primary central nervous system lymphoma (PCNSL) is a relatively uncommon and aggressive type of brain malignancy that can achieve long-term remission if it is detected promptly and treatment is initiated on time. However, it can be notorious in its presentation in atypical cases and can disguise itself as multiple other central nervous system (CNS) disorders. In this report, we describe the case of a 60-year-old immunocompetent male presenting with radiographic features, neurological deficits, and partial steroid responsiveness, which were consistent with a demyelinating disease. However, his symptomatic improvement was only transient, which prompted us to perform a biopsy. The biopsy revealed a PCNSL, and he was subsequently started on a high-dose methotrexate, rituximab, and temozolomide regimen, leading to near complete radiological remission. He underwent autologous hematopoietic stem cell transplantation post chemotherapy, which achieved durable remission with significant clinical recovery. This case highlights the risk of misdiagnosis when PCNSL presents with atypical imaging and steroid-induced improvement, while also demonstrating favourable outcomes with timely intervention with combined chemotherapy and stem cell transplantation.

## Introduction

Primary central nervous system lymphoma (PCNSL) is an uncommon form of non-Hodgkin lymphoma, which is aggressive and appears outside the lymph nodes. It makes up 2-4% of primary malignancies of the brain and can involve the brain parenchyma, leptomeninges, ocular components, or spinal cord. It is more common in elderly individuals, with a median age at diagnosis of 66 years, and in those who are immunocompromised. Despite its aggressive nature, it is a potentially curable condition if diagnosed and treated on time, even though it carries a high rate of morbidity and mortality [1]. 

Intracranial lesions are the most common site of PCNSL. Patients present with a myriad of non-specific symptoms, such as focal neurological impairments in approximately 70% of patients, neuropsychiatric symptoms in about 43% of patients, and signs of increased intracranial pressure, with only 10-20% of patients experiencing seizures [2]. 

On an MRI, PCNSL most often appears as a single, uniformly enhancing lesion that demonstrates restricted diffusion and decreased apparent diffusion coefficient (ADC) values, with MR spectroscopy revealing lower N-acetylaspartate (NAA) and higher choline peaks [3-5]. However, PCNSL can present with unusual radiological features, such as multiple lesions, absent or variable contrast enhancement, minimal diffusion restriction, and hyperintense signals on certain sequences. These unconventional presentations can closely resemble various other central nervous system (CNS) disorders, such as demyelinating conditions, metastatic disease, infectious processes, or gliomatosis cerebri, creating significant diagnostic dilemmas that may result in delayed recognition and treatment initiation [6].

Confirming the diagnosis of PCNSL is dependent on integrating characteristic radiological findings, cerebrospinal fluid (CSF) analysis findings, and histopathological confirmation through biopsy. Although CSF cytology or flow cytometry may provide data helping in reaching a diagnosis in some cases, their specificity is constrained. Therefore, a stereotactic biopsy is considered to be the gold standard for a definitive diagnosis [7,8].

This report describes a case of PCNSL with multiple, non-enhancing lesions that were initially misdiagnosed as a demyelinating disorder, thereby highlighting the critical diagnostic considerations and their therapeutic consequences.

## Case presentation

A 60-year-old immunocompetent male of Indian origin with a history of hypertension presented to the neurology outpatient department in July 2024 with complaints of headache, speech disturbances, occassional blurring of vision, right upper and lower limb weakness and occasional dysesthesias for a duration of three months. He did not have any neuropsychiatric or memory impairment. He had previously received steroid therapy with prednisolone 40 mg once a day for two months, following an MRI performed at an outside hospital, which was indicative of a demyelinating disease; however, the images and reports were unavailable. He had relapsing and remitting symptoms post this line of treatment.

**Figure 1:**
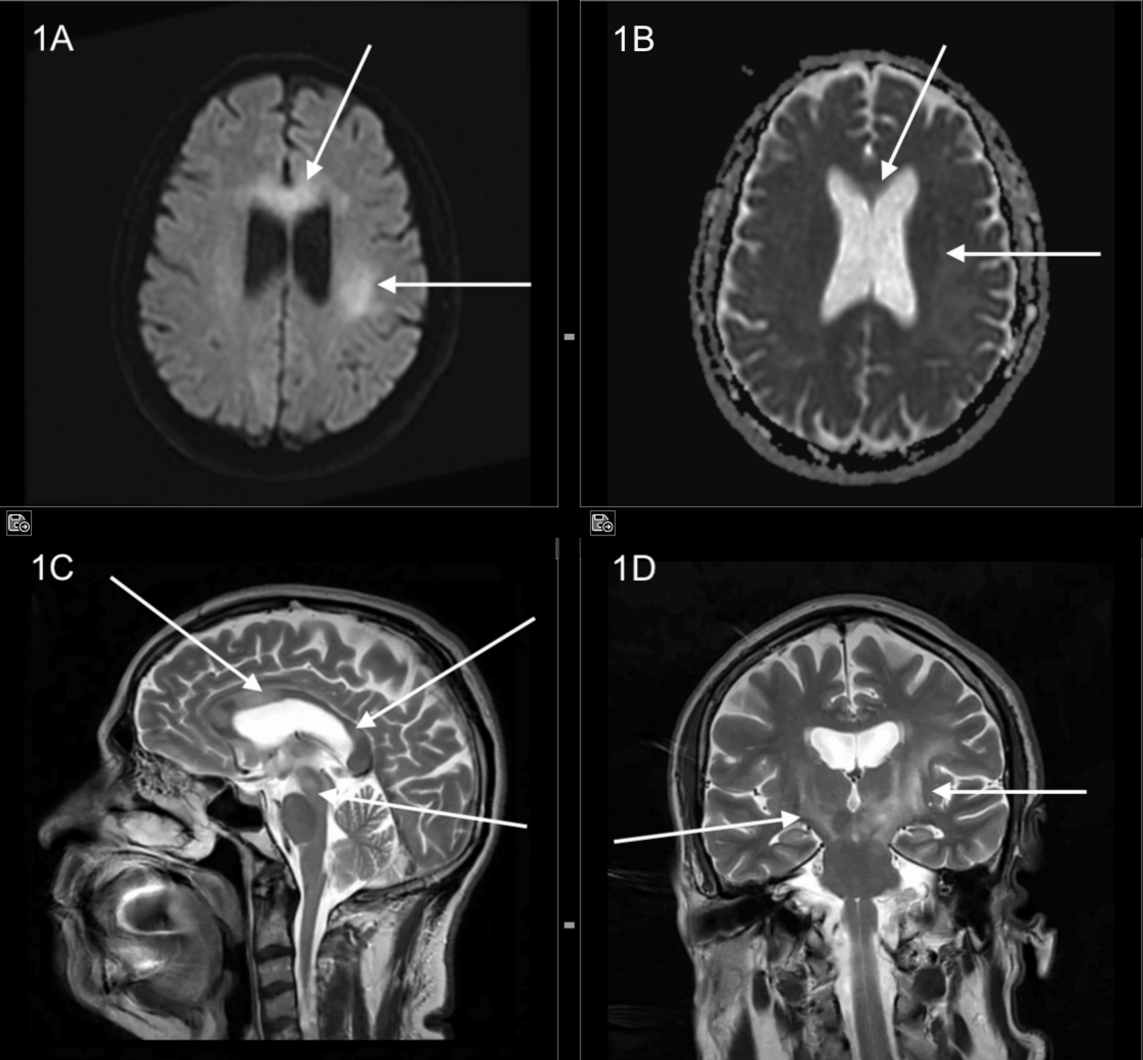
(A, B) DWI and ADC images showing DWI hyperintensity with mild reversal on ADC-weighted images in periventricular white matter and corpus callosum. (C, D) T2 sagittal (C) and T2 coronal (D): Multiple lesions showing heterogeneous signal intensity with hyperintense oedema involving corpus callosum, gangliocapsular regions, bilateral periventricular white matter, brainstem and cerebral peduncles. DWI: diffusion-weighted imaging; ADC: apparent diffusion coefficient.

**Figure 2:**
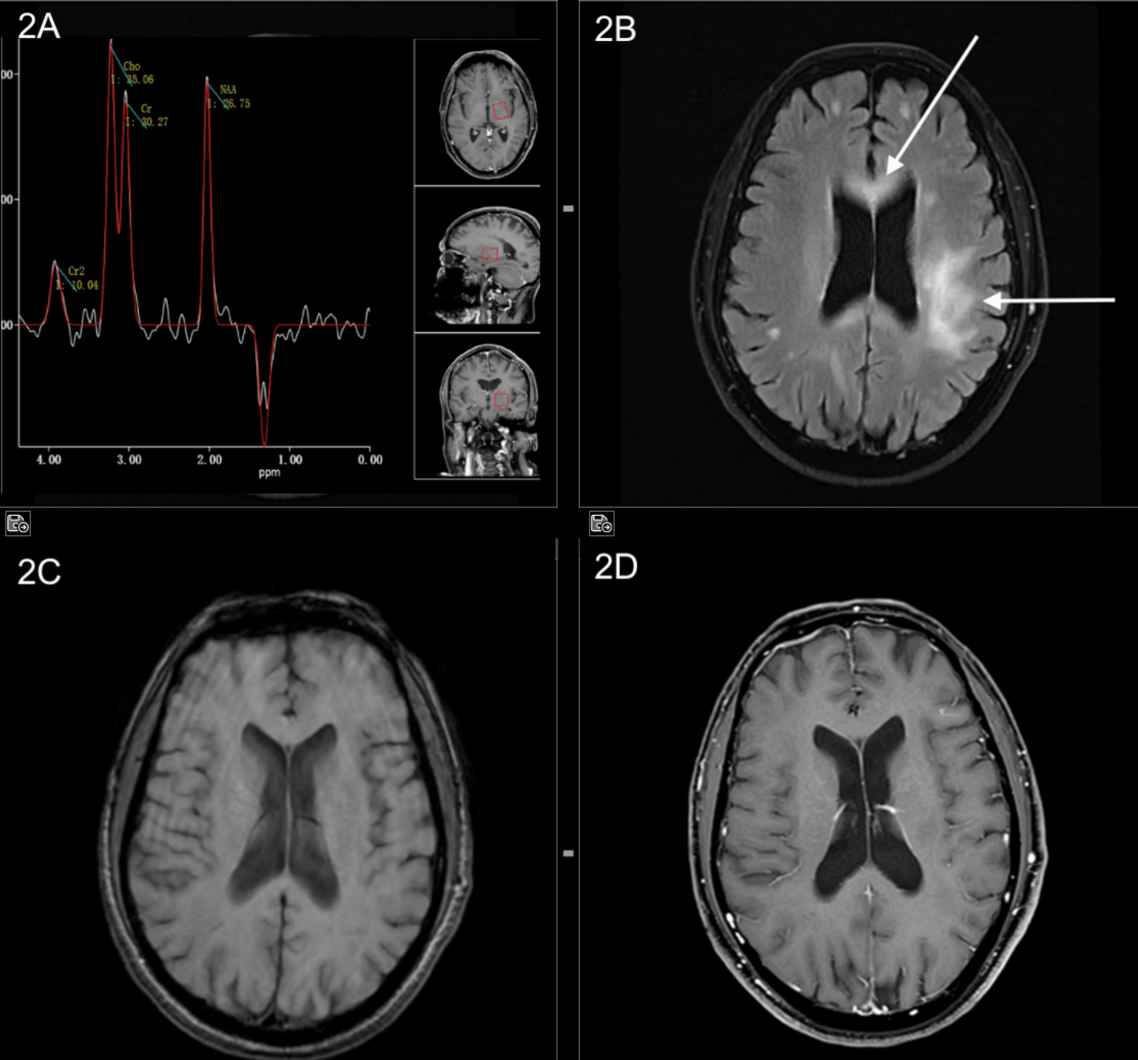
(A) MR spectroscopy: Mildly elevated choline and creatine levels, with a choline creatine ratio not more than two. NAA is preserved. (B) Axial FLAIR images showing hyperintense signal intensity in the gangliocapsular regions, periventricular and subcortical white matter and corpus callosum. (C) SWI images showing no blooming. (D) Post-contrast axial images showing no significant areas of contrast enhancement. MR: magnetic resonance; NAA: N-acetylaspartate; FLAIR: fluid-attenuated inversion recovery; SWI: susceptibility weighted imaging.

After presenting at our hospital, an MRI brain was done, which showed multiple discrete areas of T2 heterogenous signal intensity with surrounding hyperintensity involving the genu, anterior body, and splenium of the corpus callosum, right parietal lobe, left precentral gyrus, left centrum semiovale, bilateral corona radiata, left globus pallidus, posterior limbs of bilateral internal capsule, anterior limb of left internal capsule, and bilateral cerebral peduncles. No abnormal post-contrast enhancement was seen within these lesions. There was diffusion-weighted imaging (DWI) hyperintensity with mild reversal on the ADC-weighted study. Spectroscopy did not reveal any decrease in NAA (Figures 1, 2). As there was no clinical suspicion of an underlying tumour, a dedicated tumour protocol was not performed. MR spectroscopy was performed for additional information, given its cost-effectiveness compared to a perfusion study.

**Figure 3:**
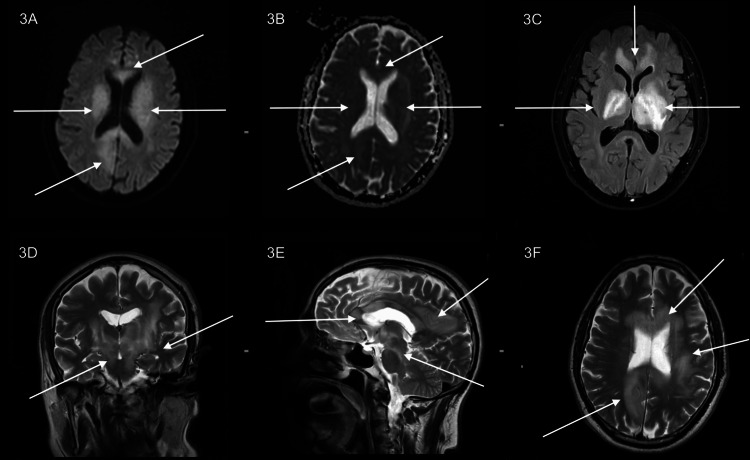
(A, B) DWI and ADC images showing diffusion restriction in bilateral gangliocapsular regions, periventricular white matter and corpus callosum. (C) Axial FLAIR images showing hyperintense signal intensity in bilateral gangliocapsular regions, periventricular white matter and corpus callosum. (D, E, F) T2 coronal, T2 sagittal and T2 axial: Multiple lesions showing heterogeneous signal intensity with hyperintense oedema involving corpus callosum, gangliocapsular regions, bilateral periventricular white matter, brainstem and cerebral peduncles. Compared to the study, as seen in Figures 1 and 2. Diffusion restriction is significantly more pronounced. There was an interval increase in the extent and size of the lesions. DWI: diffusion-weighted imaging; ADC: apparent diffusion coefficient; FLAIR: fluid-attenuated inversion recovery.

Anti-myelin oligodendrocyte glycoprotein (MOG) antibodies and aquaporin-4 antibodies were tested to exclude neuromyelitis optica spectrum disorder (NMOSD) and MOG antibody-associated disease; both results came back negative. CSF analysis, including cytology, opening pressure, oligoclonal bands, gram staining, adenosine deaminase (ADA), and acid-fast bacilli (AFB) testing, was performed, with all results negative. A comprehensive ocular examination was conducted, including slit lamp examination, fundoscopy, and perimetry, with no abnormalities detected. A visual evoked potential (VEP) study also showed normal findings. Viral markers such as Human immunodeficiency virus (HIV), hepatitis B, hepatitis C, and Epstein-Barr virus (EBV) were all negative. Based on the MRI findings and a negative CSF cytology, a demyelinating disorder was suspected, and he was started on steroid therapy with prednisolone 40 mg once a day. The patient improved symptomatically with steroid therapy and defaulted for one month due to the symptomatic improvement. He later presented to the hospital with worsening symptoms in September 2024. Given the progression of symptoms despite steroid therapy, an MRI of the brain was done, which showed multiple discrete lesions showing T2 heterogeneous signal intensity with surrounding hyperintensity and restricted diffusion, which was better appreciated in this study than in the previous one. This was noted in the left lateral aspect of the pons, midbrain, cerebral peduncles, bilateral capsuloganglionic region, a portion of the bilateral thalami, near the entire corpus callosum, bilateral peritrigonal, right posterior parasagittal fronto-parietal, left periventricular, left corona radiata, and left frontal subcortical/precentral gyral region. There was an interval increase in the extent and size of the lesions (Figure 3).

**Figure 4:**
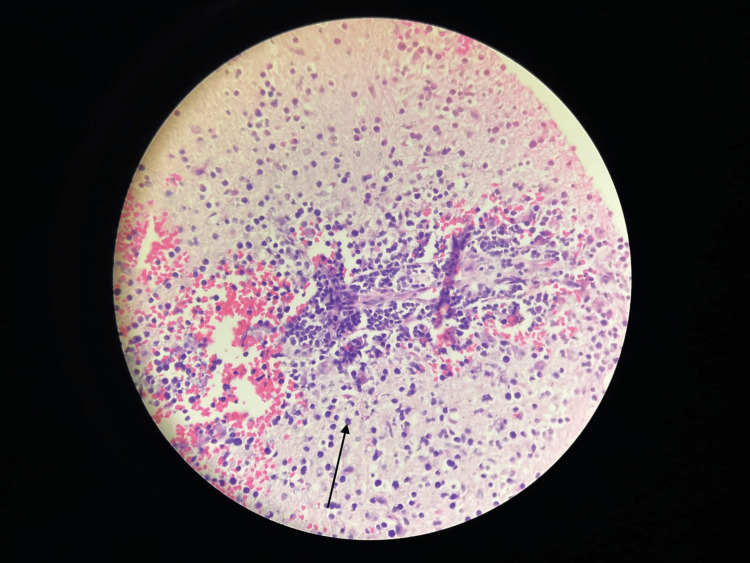
Histopathological findings. Perivascular and parenchymal infiltrate of atypical lymphoid cells.

Given the MRI findings and clinical deterioration despite therapy, a differential diagnosis of lymphoma vs glioma vs other CNS neoplasms was considered, and a stereotactic biopsy was done. The biopsy was indicative of non-Hodgkin lymphoma (Figure 4). Immunohistochemistry was suggestive of diffuse large B-cell lymphoma, germinal centre B-cell-like (GCB) subtype, with CD20, CD10, BCL6, and MUM1 positive. CSF cytology, opening pressure, and bone marrow biopsy were all normal. A positron emission tomography (PET) scan was done, which did not reveal any extracranial disease. 

He was diagnosed with PCNSL, with an International Extranodal Lymphoma Study Group (IELSG) score 2 disease (Table 1).

**Table 1:** IELSG Score IELSG: International Extranodal Lymphoma Study Group.

Variable	Observed values	Score
Age (years)	60	0
Eastern Cooperative Oncology Group Performance score	2	1
Lactate dehydrogenase, U/L	175	0
CSF protein level, mg/dl	42.3	0
Involvement of deep regions of the CNS	Yes	1
Total score		2

**Figure 5:**
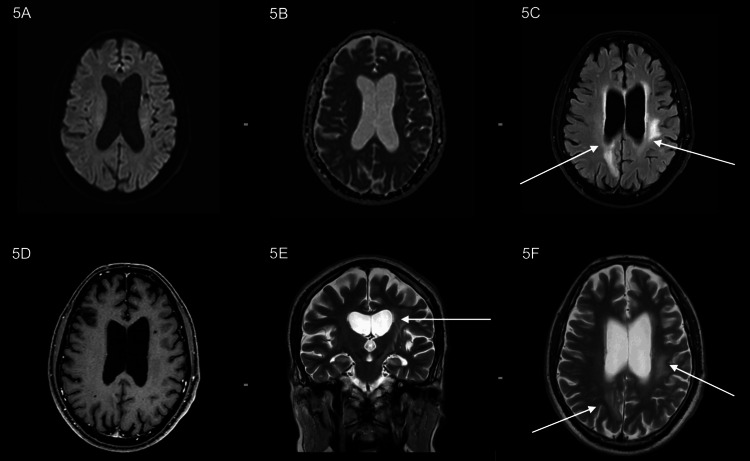
(A, B) DWI and ADC weighted images showing no significant diffusion restriction. (C) Axial FLAIR images showing hyperintensity in parietal parasagittal cortex and left periventricular white matter. (D) Axial post-contrast T1-weighted images showing no evidence of contrast enhancement. (E, F) Coronal and Axial T2 images showing T2 heterogeneous signal intensity with T2 hyperintense oedema predominantly along the left periventricular white matter, left gangliocapsular region, left cerebral peduncles and brainstem. Compared to the prior study, as seen in Figure 3: Significant interval decrease in the size and extent of lesions with resolution of diffusion restriction. DWI: diffusion-weighted imaging; ADC: apparent diffusion coefficient; FLAIR: fluid-attenuation inversion recovery.

He was subsequently started on CALGB MTR (methotrexate, rituximab and temozolamide) induction therapy from October 5, 2024, to January 22, 2025, without any grade 3 or grade 4 toxicities. This therapy was delivered in 14-day cycles. On day one of each cycle, methotrexate 8 g/m^2^ was administered; rituximab 375 mg/m^2^ was given on days three and eight during the first three cycles; and temozolomide 150 mg/m^2^ was administered from day 7 through day 11 of every odd-numbered cycle. Post-chemotherapy, he showed both clinical and radiological improvement. MRI brain in February 2025 showed near complete resolution of lesions in the corpus callosum, bilateral thalami, midbrain, with significant reduction (>80%) of lesions in the right posterior parietal, left frontal, and left corona radiata (Figure 5).

**Figure 6:**
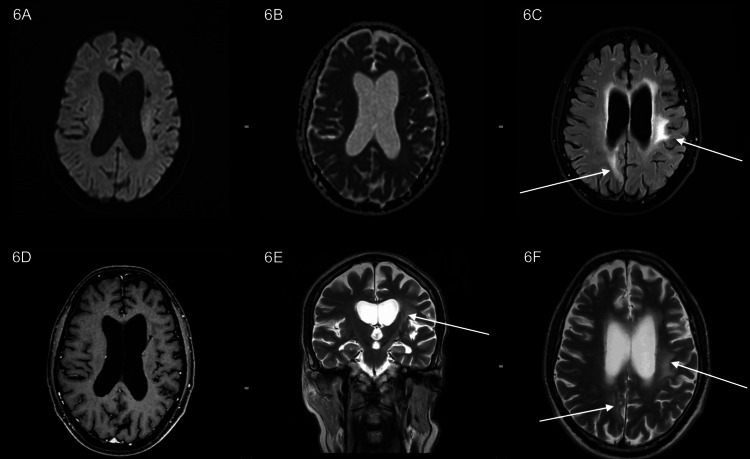
(A, B) DWI and ADC weighted images showing no significant diffusion restriction. (C) Axial FLAIR images showing hyperintensity in parietal parasagittal cortex and left periventricular white matter. (D) Axial post-contrast T1-weighted images showing no evidence of contrast enhancement. (E, F) Coronal T2 and axial T2 images showing T2 heterogeneous signal intensity with T2 hyperintense oedema predominantly along the left periventricular white matter, left gangliocapsular region, left cerebral peduncles and brainstem. Compared to the prior study, as seen in Figure 5: No significant interval changes. DWI: diffusion-weighted imaging; ADC: apparent diffusion coefficient; FLAIR: fluid-attenuation inversion recovery.

Post the induction therapy, he was planned for an autologous hematopoietic stem cell transplant. The stem cell harvest was done on February 25, 2025 with the help of a mobilisation protocol consisting of plerixafor and granulocyte colony-stimulating factor (GCSF). He underwent a conditioning regimen with thiotepa 5 mg/kg on Day -5 and Day -4 and carmustine at 400 mg/m^2^ on Day -6. Stem cell infusion was done on March 6, 2025 (Day 0). On March 15, 2025 (Day +9 post-transplant), platelet and neutrophil engraftment was achieved. He had grade 3 mucositis and culture-negative febrile neutropenia during the post-transplant course. A response MRI brain was done 100 days post transplant, which demonstrated a maintained response to treatment (Figure 6).

## Discussion

Detecting PCNSL can be particularly elusive when its clinical or radiographic features diverge from the typical presentation [6]. Relying solely on clinical assessment cannot separate PCNSL from other CNS conditions, as the non-specific symptoms in PCNSL are also seen in cases of demyelinating disorders, and other inflammatory or infectious processes [1,6].

When we came across this 60-year-old patient, his presentation seemed straightforward as a classic case of demyelinating disease. The MRI findings, which, when combined with his neurological symptoms and initial response to steroids, suggested a demyelinating disease. However, this case demonstrated that PCNSL may closely resemble several other CNS disorders, especially when treated with steroids prior to imaging. What stood out the most about this patient was how convincingly his imaging resembled an inflammatory demyelinating disease. Instead of the characteristic imaging findings that are normal to expect in PCNSL, we found ourselves looking at multiple areas with a T2 hyperintense signal without contrast uptake and only subtle ADC reversal, resembling demyelination or gliomatosis cerebri. Such unconventional imaging with absent or variable enhancement, minimal diffusion restriction, and normal spectroscopy surely occurs in a minority of cases and frequently leads to misdiagnosis [6].

The absence of anti-MOG and aquaporin-4 antibodies, normal oligoclonal bands, and unremarkable CSF studies created a diagnostic scenario that directed us towards inflammatory demyelination rather than neoplasia. It is worth mentioning that, as reported in literature, CSF cytology detection rates are 2-32%, often yielding false negatives when the tumour burden is low or after steroid pre-treatment [9,10]. 

The patient’s symptomatic improvement following steroid treatment further complicated differentiation. However, this clinical improvement corresponds to a well-documented phenomenon where at least 40% of PCNSL patients experience transient symptom relief with steroids, which can temporarily shrink lesions and obscure histopathology [6,9]. Administering steroid therapy before obtaining tissue for histopathology must be avoided whenever possible [9]. In our patient, there is a possibility that pre-treatment with steroids led to a false negative radiographic picture and CSF analysis, which led to a diagnosis of a demyelinating disorder. Further, the patient’s symptomatic relief prompted them to forgo follow-up, resulting in a delayed diagnosis.

Despite steroid therapy, the patient returned with worsening symptoms, and we knew we had missed something important. Follow-up MRI showed progression and persistence of the lesions, with more pronounced diffusion restriction than before. This, along with clinical deterioration, forced us to reconsider our initial diagnosis and pursue tissue confirmation through stereotactic biopsy. Histopathology confirmed diffuse large B-cell lymphoma of the germinal centre B-cell-like (GCB) subtype (CD20, CD10, BCL6, and MUM1 positive), consistent with IELSG score 2 disease [11,12].

This diagnosis allowed initiation of high-dose methotrexate-based chemotherapy combined with temozolomide and rituximab (CALGB MTR), which is shown to achieve complete remission in about two-thirds of cases [13]. In this case, our patient underwent the induction therapy, post which he had a near-complete remission of the disease. Long-term outcomes further improve with autologous haematopoietic stem cell transplantation (ASCT) after high-dose chemotherapy, which has reported an overall survival and disease-free survival of 77% at a follow-up of approximately 25 months duration in one study [14], and 69% progression-free survival in one study [15]. The sustained remission observed in our patient at a follow-up of 100 days post transplant, following chemotherapy and ASCT, aligns with these reported outcomes.

## Conclusions

This case highlights several important clinical lessons regarding the diagnostic complexity of PCNSL, particularly when it presents with atypical radiographic features, negative CSF cytology, non-specific symptoms and steroid-responsive symptoms resembling demyelinating disorders. Clinicians should maintain a broad differential diagnosis at initial presentation, especially because symptoms are often non-specific. When clinical improvement is not sustained, worsens, or when initial diagnostic tests are inconclusive or possibly affected by prior treatments with steroids, prompt reassessment of the diagnosis is crucial.

During reassessment, repeat investigations such as imaging and biopsy should be considered. In this case, that reconsideration led to a life-saving diagnosis and ultimately to a sustained remission at follow-up of 100 days post transplant. This patient's journey from misdiagnosis to successful treatment, from presenting to the hospital in a wheelchair to being able to walk independently, further emphasises the importance of timely diagnosis and initiation of therapy.
